# Evidence for the Dissemination to Humans of Methicillin-Resistant *Staphylococcus aureus* ST398 through the Pork Production Chain: A Study in a Portuguese Slaughterhouse

**DOI:** 10.3390/microorganisms8121892

**Published:** 2020-11-29

**Authors:** Ons Bouchami, Maria João Fraqueza, Nuno Alexandre Faria, Valquíria Alves, Opeyemi Uwangbaoje Lawal, Herminia de Lencastre, Maria Miragaia

**Affiliations:** 1 Laboratory of Bacterial Evolution and Molecular Epidemiology, Instituto de Tecnologia Química e Biológica (ITQB-NOVA) António Xavier, 2780-157 Oeiras, Portugal; onsbouchami@itqb.unl.pt (O.B.); nfaria@itqb.unl.pt (N.A.F.); opeyemi@itqb.unl.pt (O.U.L.); 2 Laboratory of Molecular Genetics, Instituto de Tecnologia Química e Biológica (ITQB-NOVA) António Xavier, 2780-157 Oeiras, Portugal; lencash@mail.rockefeller.edu; 3 Centre for Interdisciplinary Research in Animal Health (CIISA), Faculdade de Medicina Veterinária, Universidade de Lisboa, 1300-477 Lisboa, Portugal; mjoaofraqueza@fmv.ulisboa.pt; 4 Serviço de Patologia Clínica, Hospital Pedro Hispano, 4464-513 Senhora da Hora, Portugal; valquiria.alves@ulsm.min-saude.pt; 5 Laboratory of Microbiology and Infectious Diseases, The Rockefeller University, New York, NY 10065, USA

**Keywords:** MRSA, ST398, slaughterhouse, pork chain production, transmission, invasive disease

## Abstract

Livestock-associated methicillin-resistant *Staphylococcus aureus* (LA-MRSA) ST398 was recovered from infections in humans exposed to animals, raising public health concerns. However, contact with food producing chain as a means of transmission of LA-MRSA to humans remains poorly understood. We aimed to assess if pork production chain is a source of MRSA ST398 for human colonization and infection. MRSA from live pigs, meat, the environment, and slaughterhouse workers were analyzed by Pulsed-Field Gel Electrophoresis (PFGE), *spa*, MLST typing, SNPs and for antibiotic resistance and virulence gene profiles. We compared core and accessory genomes of MRSA ST398 isolated from slaughterhouse and hospital. We detected MRSA ST398 (t011, t108, t1451) along the entire pork production chain (live pigs: 60%; equipment: 38%; meat: 23%) and in workers (40%). All MRSA ST398 were multidrug resistant, and the majority carried genes encoding biocide resistance and enterotoxins. We found 23 cross-transmission events between live pigs, meat, and workers (6–55 SNPs). MRSA ST398 from infection and slaughterhouse environment belonged to the same clonal type (ST398, t011, SCC*mec* V), but differed in 321–378 SNPs. Pork production chain can be a source of MRSA ST398 for colonization of human slaughterhouse workers, which can represent a risk of subsequent meat contamination and human infection.

## 1. Introduction

Antimicrobial resistance threatens prevention and treatment strategies of an increasing range of bacterial infections. One of the bacteria for which new antibiotics are urgently needed due to multidrug resistance is methicillin-resistant *Staphylococcus aureus* (MRSA). After the emergence and spread of MRSA in the nosocomial environment and the healthy community [1], MRSA was also frequently described in animal production environments colonizing animals and humans in close contact with animals, such as veterinarians and farm workers [2,3,4], so-called livestock-associated MRSA (LA-MRSA). The emergence of MRSA in farms has been attributed to the intensive and misuse of antibiotics, including beta-lactams, in animal husbandry and treatment of human bacterial infections [5]. This together with the inadequate management of residues have increased antibiotic pollution in the environment promoting the selection, dissemination and emergence of MRSA [6]. Our own studies have demonstrated that the development and dissemination of the beta-lactam resistance determinant among human and animal commensals was promoted by the prolonged and abusive use of beta-lactams [7,8,9].

Besides direct contact with animals, another possible route of dissemination of LA-MRSA to humans is through the food production chain either through food manipulation, if not handled correctly, and consumption, if meat is not properly cooked. Actually, many studies have already confirmed the contamination of several food products with MRSA [10] that once spread to humans can cause food poisoning, which is one of the most common food-borne diseases, and eventually, death, mainly in immunocompromised patients in hospitals [11]. However, no study has ever provided evidence that MRSA contaminating food was the origin of nosocomial infections.

LA-MRSA strains worldwide were predominantly assigned to clonal complex (CC) 398 [12] a lineage that was described to be multiresistant, namely to penicillin (*blaZ–blaI–blaR*), tetracycline (*tet*(M) and *tet*(K)), macrolide–lincosamide–streptogramin B (MLSB) (*erm*(A), *erm*(B), and *erm*(C)), lincosamide (*lnu*(A)), and aminoglycosides (*aacA*–*aphD*, *aadD*) [13]. Besides, LA-MRSA CC398 can also carry several virulence genes, such as hemolysin-encoding genes (α and δ hemolysin, *hlb* genes), leukotoxin Panton-Valentine (*lukPV* genes), staphylococcal enterotoxins (SEs, *se* genes), exfoliatins (ET, *et* genes), leukotoxins, and toxic shock syndrome toxin (TSST-1, *tst* gene) [14]. CC398 has been recovered worldwide as colonizers of different animal species including bovine, avian, equine, poultry, horse, veal calves and pigs [15,16], but has also been described to colonize humans [15,16,17,18] and as a cause of infections in hospitals [18]. Studies based on whole genome sequencing (WGS) have shown that most ST398 in humans are MSSA [19]. In contrast, in animals, ST398 is usually associated with MRSA [20]. However, cases of MRSA CC398 in humans in close contact with production animals have been increasing rapidly in some countries such as in the Netherlands, where it reaches up to 25% [18,21] and also in Europe, Asia, and the United States [21], constituting a public health concern. Most of human ST398 MSSA infections are caused by *spa* type t571 strains, whereas the predominant *spa* types among pig-associated MRSA isolates are t011 and t108 [20], suggesting that the transmission of animal associated MRSA strains to humans is not frequent.

Portugal is one of the European countries with the highest rates of nosocomial MRSA [22], reaching 50% in 2017; although a decrease in prevalence has been noticed in recent years, the problem is far from being solved. Periodic surveillance of MRSA in Portugal has shown that EMRSA15 has been the main clone in hospitals for the last 15 years [23]. While ST398 MRSA was found in high prevalence in live pigs in Portuguese farms [24], to our knowledge, no ST398 MRSA has been identified colonizing healthy humans or causing infections in Portuguese hospitals.

In this study, we aimed to assess if LA-MRSA could be transmitted along a pork production chain and whether this could constitute a risk of human colonization and a cause of infection in hospitals.

## 2. Material and Methods

### 2.1. Ethical Considerations

Formal ethical approval was not required since non-invasive procedures were used and no animal tissues were collected. Animal isolates originated from the skin of pigs and from rectal swabs collected according to International Organization for Standardization (ISO) guidelines for non-destructive carcass sampling. Moreover, samples were obtained as part of slaughterhouse safety and good hygiene processing control procedures.

Human isolates were collected from slaughterhouse workers as part of good hygiene practice verification procedures. Nosocomial bacterial isolates were obtained as part of routine surveillance and were analyzed anonymously. All data was collected according to European Parliament and Council decision for the epidemiological surveillance and control of communicable disease in the European community [25,26]. Ethical approval and informed consent were thus not required. Manipulation of the isolated bacteria was performed in conditions that guarantee research staff safety.

### 2.2. Sample Collection

In total, 75 samples were collected in 2016 from a slaughterhouse in metropolitan area of Lisbon, Portugal, at two time points (summer and winter). The slaughterhouse is dedicated to the abattoir of both bovine and pigs, but to each animal species a completely separate production line is attributed. A total of 150 pigs are slaughtered per hour and pigs originate mainly from slaughterhouse—associated farms, in a vertical management approach, which are all national from the Center and the South of Portugal. Additionally, the slaughterhouse provides the abattoir service to other farms in Portugal and can receive pigs from other countries, mostly from Spain. Samples were collected at slaughterhouse entrance from 20 live pigs: 10 animals sampled in summer (1st sampling period), only in the ear (*n* = 10) and 10 other animals sampled in winter (2nd sampling period) in both ear (*n* = 10) and rectum (*n* = 10). Additionally, samples were collected from: the animals respective pork pieces (belly bone in rind on, *n* = 10; shoulder bone in rind off, *n* = 10; trimmings, *n* = 7); the clean and dirty equipment in contact with the meat (conveyor from deboning line, *n* = 4; cutting table (*n* = 4); and the operators involved in the bleeding step after pigs insensibilization and carcass deboning/cutting room of production process (dirty hands, *n* = 5; clean hands, *n* = 5) (~20% of the number of operators involved in the pig slaughterhouse line and meat cutting process). For pig ears, meat pieces, worker’s hands, conveyor and cutting table, samples were collected using 10 × 10 cm sterile cotton gauze humidified with 0.9% NaCl, which were thereafter enriched in peptone water solution at 37 °C for 24 h. The area for pieces and equipment was 500–1000 cm^2^ defined with a 10 × 10 cm plastic template and according to the guidelines of ISO 18593:2004. Samples from pig rectum were collected with a sterile swab and enriched in the same conditions. After overnight enrichment, samples were conserved in TSB with 15% glycerol at −72 °C.

### 2.3. Bacterial Isolation

Enriched samples (100 µL) were spread over the surface of a chromogenic selective media for MRSA, CHROMagar MRSA (CHROMagar, Paris, France) [27] using sterile glass beads and incubated at 37 °C for 24 h to 48 h. The growth of any mauve colony was considered to be positive, indicating MRSA. Coagulase testing was performed for all presumptive MRSA (mauve-colored colonies) by latex agglutination assay using the Staphaurex™ Plus kit (Remel—Oxoid, Madrid, Spain). One MRSA isolate per sample was analyzed. An ST398-Vc (5C2&5) MRSA isolate (SRL354), collected from an infection blood sample in a Portuguese hospital in 2016 in the same geographic region, was also included for comparison.

### 2.4. Species Identification

*S. aureus* isolates were confirmed at the species level by PCR amplification of the *nuc* gene [28]. Isolates with ambiguous results were identified by PCR amplification and sequencing of the *tuf* gene [29].

### 2.5. Antimicrobial Susceptibility Testing

Antimicrobial susceptibility testing was performed using the disk diffusion method on Muller-Hinton agar (Difco, Detroit, MI, USA) according to the recommendation to the guidelines of the European Committee on Antimicrobial Susceptibility Testing (EUCAST, 2017) (http://www.eucast.org/, accesed on 9 January 2017) [30]. A total of 17 antibiotics (Oxoid, Basingstoke, UK) were tested: oxacillin (OXA, 1 μg), cefoxitin (FOX, 30 μg), penicillin (P, 10 UI), gentamicin (CN, 10 μg), ciprofloxacin (CIP, 5 μg), erythromycin (E, 15 μg), clindamycin (DA, 2 μg), chloramphenicol (C, 30 μg), rifampin (RD, 5 μg), tetracycline (TE, 30 μg), vancomycin (VA, 30 μg), Teicoplanin (TEC, 30 μg), trimethoprim-sulfamethoxazole (SXT, 1.25/23.75 μg), quinupristin/dalfopristin (QDA, 15 μg), fosfomycin (FOS, 50 µg), linezolid (LNZ, 30 µg) and fusidic acid (FD, 10 µg).

The minimum inhibitory concentrations (MICs) for oxacillin and cefoxitin were determined by E-test (AB-biodisk, Dalvogen, Sweden) on Muller-Hinton agar (Difco, Detroit, MI, USA) and interpreted as recommended by EUCAST [30].

Isolates that presented resistance to three or more classes of antibiotics, other than beta-lactams, were classified as having a multidrug resistance profile [31].

### 2.6. DNA Preparation

Genomic DNA of pure cultures for PCR was extracted by the boiling extraction method as previously described [32]. Guanidine isothiocyanate extraction method [33] was used to extract chromosomal DNA of control strains: USA300, ATCC31890, MW2, TY114, FRI913, RIMD31092, NCTC7428, TC-7, TY-4, ATCC49775, FRI569, and FRI472 were used for the detection of virulence genes (including super-antigenic toxins, hemolysins, and leukocidins) and COL, CV479, CV485, HPV107, and FRI569 were used for the detection of biocides.

### 2.7. Detection of Antimicrobial Resistance Genes

The presence of the *mecA* and *mecC* genes was detected by PCR amplification as previously described [34,35]. The presence of the efflux pump genes responsible for biocide resistance was determined by PCR amplification of *qacAB*, *smr*, *norA*, *lmrS*, *mepA*, and *sepA* genes according to Floyd et al. [36], Noguch et al. [37] and Couto et al. [38].

### 2.8. SCCmec Typing

The structure of the SCC*mec* was determined by a multiplex PCR using specific primers to each SCC*mec* type [39]. If isolates were non-typeable by this method, SCC*mec* typing was performed based on the amplification of the *mec* and *ccr* complexes as described by Okuma et al. [28]. SCC*mec* types were classified using the guidelines proposed by the International Working Group on the classification of Staphylococcal Cassette Chromosome Elements (IWG-SCC) [40].

### 2.9. spa Typing

*spa* typing was performed by a PCR amplification and sequencing of a polymorphic region of the *S. aureus* protein A gene (*spa* gene) [41]. The *spa* types were assigned using the RIDOM web server (v. 2.2.1, http://spaserver.ridom.de, accessed on 20 March 2015).

### 2.10. Detection of Toxins and Virulence Genes

The presence of leucocidins (*lukF*-*lukS*-PV [Panton–Valentine leucocidin (PVL) determinant], *lukDE* and *lukM*), hemolysins *(hlb*, *hlg* and *hlgv*) and super-antigenic toxins (*eta*, *etb*, *etd*, *sel* and *sep*) were detected by PCR [42]. The presence of pyrogenic toxins (PTs) was performed by PCR amplification of *tst* gene, which encodes toxic shock syndrome toxin (TSST) and *sea*, *seb*, *sec*, *sed*, *see*, *seg*, *she*, *sei*, and *sej* genes encoding the staphylococcal enterotoxins (SEs) as previously described by Monday et al. [43]. Arginine catabolic mobile element (ACME) allotypes (type I to III) were defined based on multiplex PCR amplification using primers specific to the loci *arc* and *opp3*, as previously described [44].

### 2.11. Immune Evasion Cluster (IEC) Cluster Detection

The presence of IEC genes (*sak*, staphylokinase; *chp*, chemotaxis inhibitory protein; *scn*, staphylococcal complement inhibitory protein; prophages of integrase group 3 (φSaint3) which are known to be carried by β-hemolysin converting bacteriophages in human isolates, was detected by PCR using the same set of primers previously described by van Wamel et al. [45] and Goerke et al. [46].

### 2.12. Pulsed-Field Gel Electrophoresis (PFGE)

PFGE for MRSA was carried out as described previously [47,48] using SmaI and Cfr9I. PFGE restriction band patterns were analyzed automatically using BioNumerics Software (v. 4.5) from Applied Maths (Sint-Martens-Latem, East Flanders, Belgium) [49] with previously defined parameters: optimization of 0.5% and position tolerance of 1.25% [49]. Dendrograms were constructed by the unweighted-pair group method using average linkages (UPGMA) and PFGE types and subtypes defined by groups formed at 80% [50] and 95% Dice similarity cutoffs, respectively, as previously defined [49]. Two strains belonging to the same PFGE subtype were considered to belong to the same chain of transmission as previously suggested [51].

### 2.13. Whole Genome Sequencing and Bioinformatics Analyses

Genomic DNA was isolated from four strain pairs: ZPP1/ZA8, ZM2/ZA6, ZPA12/ZA3 and ZP25/ZP11 belonging to the same PFGE subtypes and collected from different sources, using the Qiagen DNeasy Blood & Tissue Kit (Qiagen, Limburg, The Netherlands) and sequenced by Illumina NextSeq 500 system (San Diego, CA, USA). Libraries for genome sequencing were constructed using the Nextera XT DNA sample preparation kit (IIIumina) and sequenced using 150 bp pair-end reads with an estimated coverage of 120×. After trimming, the reads were *de novo* assembled into contigs using the CLC Genomics Workbench 9.0 (Qiagen, Hilden, Germany) analysis package with default parameters. The human ST398-Vc (5C2&5) MRSA isolate (SRL354), collected from an infection blood sample in a Portuguese hospital, was sequenced according to the protocol described, with the following alterations: sequencing was performed in Illumina NextSeq using 150 bp paired-end reads with a estimated coverage of 100×. Raw data was assembled using INNUca v. 3.1 pipeline [52].

The assembled contigs of the nine representative strains were analyzed for the presence of acquired antimicrobial resistance and virulence genes by ABRicate v. 0.8.7 (https://github.com/tseemann/abricate, accessed on 14 September 2018) using ABRicate’s ResFinder [53], CARD [54], VFDB [55] databases (accessed 28 July 2019). A percentage identity of 90% and coverage of 90% of the respective gene length were considered to be positive result. Plasmids were identified using the PlasmidFinder server v. 2.1 (https://cge.cbs.dtu.dk/services/PlasmidFinder/, database 2 April 2020) [56]. SCCmecFinder v. 1.2 (https://cge.cbs.dtu.dk/services/SCCmecFinder, database 5 February 2020) [57] was used for the detection of SCC*mec* type.

### 2.14. Core Genome Single Nucleotide Polymorphisms (SNPs) and Genomic Characterization

SNPs were identified separately within each strain, using CSI Phylogeny v. 1.4 (https://cge.cbs.dtu.dk/services/CSIPhylogeny) pipeline [58], available at the Centre of Genomic Epidemiology (CGE) of the Technical University of Denmark (DTU). Mapping of *de novo* assembled contigs against the ST398 reference genome (Strain ISU926; GenBank accession number CP017091) [59] was carried out using BWA v0.7.2 [60]. Single nucleotide polymorphisms (SNPs) were identified on the basis of the mpileup files generated by SAMTools v. 0.1.18 [61]. The criteria used for calling SNPs were as follows: a minimal relative depth at SNP positions of 10%, a minimal Z-score of 1.96, a minimal SNP quality of 30 and a minimal read mapping quality of 25. The minimum distance between SNPs was disabled and all indels were excluded. An alignment of the SNPs was then created by concatenating the SNPs based on their position on the reference genome.

Gubbins v. 2.4.1 pipeline was run using default settings to detect the recombinant regions in each of the nine full genome alignments based on the SNP density [62]. The polymorphic sites resulting from recombination events were first detected and filtered out. The filtered SNP output was transformed into SNP distance matrix using the snp-dists v. 0.62 (https://github.com/tseemann/snp-dists). Maximum likelihood trees were reconstructed from concatenated SNPs (from the alignment) by RAxML and visualized using MEGA 7 software [63] and Microreact [64]. Strains were considered to belong to the same direct chain of transmission if they had less than 50 SNPs difference as previously suggested [65].

### 2.15. Statistical Analysis

Statistical analyses were performed using GraphPad Prism v. 8.0 (GraphPad Software Inc., San Diego, CA, USA). The Chi-square test was used to analyze the differences between groups. A *p* value < 0.05 was considered statistically significant.

## 3. Results

### 3.1. MRSA Were Prevalent in Pigs and the Entire Slaughterhouse Environment

A total of 75 samples were collected in the slaughterhouse in two time periods (summer and winter) out of which 41% (31/75) were positive for MRSA (1st sampling: 14/32; 44% and 2nd sampling 17/43; 39.5%). Every type of site sampled was colonized or contaminated with MRSA. The highest prevalence was observed in live pigs (18/30, 60%), wherein ear was a richer MRSA reservoir than the rectum (67% and 50%, respectively, *p* < 0.1). MRSA was also found in the clean and dirty hands of operators (4/10, 40%), and clean conveyor from deboning line and dirty cutting table (3/8, 38%). Although with the lowest MRSA frequency among all samples, the prevalence of MRSA in meat reached as high as 23% (6/26).

### 3.2. MRSA from Pigs and Slaughterhouse Belonged to a Diverse Group of Strains Belonging to ST398

The total DNA of all the isolates was resistant to digestion by SmaI, suggesting that isolates belonged to the LA-MRSA ST398. The clonal type of these 31 MRSA isolates was further confirmed by the fact that the ST398 MRSA control strain (NCTC 2825) belonged to the same PFGE type as the isolates analyzed. Furthermore, the three *spa* types detected among the isolate collection were closely related and previously described to be associated with MRSA-ST398 (t011 (58%), t108 (39%) and t1451 (3%)) [66]. Analysis of whole genome sequence data of eight strains belonging to different PFGE types (strains ZPP1, ZA8, ZM2, ZA6, ZPA12, ZA3, ZP25 and ZP11) allowed confirming that they belonged to ST398. The digestion with SmaI isoeschizomer, Cfr9I, originated a single PFGE type and 14 genetically related subtypes among the 31 isolates analyzed in this study, revealing a relatively high heterogeneity of the bacterial collection analyzed (Figure S1).

### 3.3. MRSA ST398 in the Slaughterhouse Were Multidrug Resistant

The SCC*mec* type V was the most common type in the ST398 MRSA isolates from this study being found in 55% of isolates (17/31), mainly among strains belonging to *spa* type t011 (15/17 88%). The remaining isolates carried either a variant of SCC*mec* type V (8/31, 26%), carrying *mecA*, *ccrC* and the J3 region of SCC*mec* type III, or were non typeable (6/31, 19%).

Besides being resistant to all beta-lactams, due to the presence of SCC*mec*, the MRSA isolates tested showed high rates of phenotypic resistance to tetracycline (31/31, 100%), clindamycin (31/31, 100%), erythromycin (23/31, 76%), gentamicin (19/31, 61%) and chloramphenicol (20/31, 65%). The resistance rates to quinupristin/dalfopristin (5/31, 16%) and ciprofloxacin (4/31, 13%) were low.

There were no isolates resistant to fosfomycin, teicoplanin, vancomycin, linezolid, rifampicin, fusidic acid and trimethoprim-sulfamethoxazole. All MRSA isolates tested showed a multidrug resistance profile. The most predominant resistance profile included resistance to penicillin, oxacillin, cefoxitin, erythromycin, clindamycin, tetracycline, and chloramphenicol (5/31, 16%). Furthermore, all the MRSA isolates showing this profile of resistance were from live pigs.

Actually, we observed that the rates of resistance to penicillin, oxacillin, cefoxitin, gentamicin, erythromycin, clindamycin, tetracycline, chloramphenicol, and quinupristin/dalfopristin were significantly higher (*p* < 0.001) in live pigs at the slaughterhouse entrance than in the remaining sampling sites inside the slaughterhouse, suggesting that resistance to these antibiotics was acquired by *S. aureus* before entrance into the slaughterhouse environment. However, the rates of resistance to ciprofloxacin were significantly higher in meat compared to live pig, operator, and processing equipment (*p* < 0.001). This was probably related to the presence of genes encoding efflux pumps, previously reported to also transport quinolones [38] that were significantly more common in ciprofloxacin-resistant isolates compared to ciprofloxacin-susceptible ones isolated from meat (*p* < 0.0001 for genes *lmrs*, *norA* and *qac*; *p* = 0.0083 for *mepA* and *sepA*). Moreover, each ciprofloxacin resistance isolate, carried at least four genes encoding efflux pumps, whereas ciprofloxacin sensitive isolates had only one or three genes.

Noteworthy, the prevalence of biocide resistance genes was extremely high namely for *mepA* (87%), *sepA* (71%), *norA* and *lmrS* (61% each), genes coding for biocide efflux pumps, and *qacAB* (52%) conferring resistance to quaternary ammonium compounds (QACs). The *smr* gene, which belongs to the small multidrug resistance (SMR) family was absent in all MRSA isolates studied. A marked difference in the distribution of biocide resistance genes among different sampling sites was observed with highest frequency detected in workers (57–100%) (*p* < 0.01). This is in accordance with the observation that the frequency of MRSA ST398 in both clean and dirty hands was very similar (43% and 40%, respectively).

### 3.4. MRSA ST398 in the Slaughterhouse Had a High Pathogenic Potential

Besides being multidrug resistant, ST398 MRSA isolates carried several virulence genes, namely *hlb* and *hlg*, encoding hemolysins (65% each) and *sel*, *sep* and *sea*, encoding staphylococcal enterotoxins (65%, 55% and 3%, respectively). All the isolates were negative for leukotoxins, toxic shock syndrome toxin, ACME and PVL. The MRSA isolates displayed four different virulence profiles with considerable variability. Among all profiles, the genotype *hlb hlg* (67%) was the most frequent.

We also observed a differential distribution of toxins among the different sampling sites. In particular, toxins were mainly and significantly (*p* < 0.001) enriched in isolates collected from workers’ hands (100%), when compared to live pigs, processing equipment (58.5%, each), and meat samples (54.25%).

The immune evasion gene cluster (IEC), a virulence determinant typical for *S. aureus* of human origin that is usually absent in *S. aureus* of animal origin [67] was lacking from all the 31 MRSA ST398 isolates collected from animals, as well as from the unique nosocomial MRSA ST398 included in the study. The results suggest that all of the isolates belonged to MRSA that had origin in pigs.

### 3.5. Evidence for the Dissemination of MRSA ST398 along the Food Production Chain

**Figure 1:**
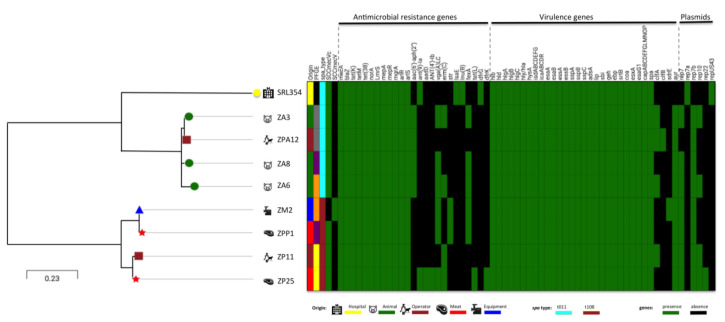
Comparison between hospital and slaughterhouse isolates based on single nucleotide polymorphisms (SNPs) identified through CSIPhylogeny v1.4 analysis. SNPs tree visualization was performed with Microreact server. Green boxes indicate the presence of acquired antimicrobial resistance genes, virulence genes or the molecular characteristic indicated in the Figure. Black boxes indicate the absence of genetic determinants.

Twenty-three isolates shared the same PFGE subtype suggesting the occurrence of at least 23 events of cross-transmission of ST398 within the slaughterhouse environment and food production chain (Figure S1) (See below WGS analysis). To confirm this hypothesis, four pairs of isolates with similar PFGE subtypes were further analyzed and compared by whole genome sequencing. The relatedness between these four pairs of strains was determined by phylogenetic analysis based on SNPs. The genome sequences of the eight isolates were mapped and aligned against the reference sequence for ISU926 ST398 [59] and SNPs were called. The percentage of reference genome covered by all isolates was 94% implying that 2664,700 positions were found in all analyzed genomes. The SNPs arising from recombination events were filtered out using Gubbins pipeline and the output was used to construct SNP distance matrix using the snp-dists pipeline. A total of 245 qualified SNPs was used to construct the maximum likelihood tree. The more similar pairs of isolates corresponded to equipment-meat (ZM2-ZPP1, six SNPs), followed by the pair live pig-worker (ZA6-ZPA12, 28 SNPs), pig-pig (ZA3/ZA8, 46 SNPs) and meat-worker (ZP25-ZP11, 55 SNPs) (Figure 1). All the pairs belonging to the same PFGE subtype and selected for SNP analysis, except one, appear to correspond to direct transmission events. Although ZP25-ZP11 pair does not comply with the criteria established for the occurrence of a direct transmission (<50 SNPs), the number of SNP differences was very near the cut-off (55 SNPs). Results suggest that the 23 isolates that share the same PFGE subtype with at least another isolate in the collection, are probably part of specific chains of transmission.

Besides differing in a small number of SNPs in their core genome, the four pairs of strains appear to be similar also at the level of the accessory genome since all the isolates carried similar antibiotic resistance, virulence, biofilm, and biocide genes content as well as plasmids as defined by WGS data analysis (Table S1). In particular, the pair of the highly related isolates (six SNPs) from meat and equipment were indistinguishable regarding their antibiotic resistance, virulence and plasmids profile and shared the same SCC*mec*, *spa* and MLST type. Overall the results suggest the existence of frequent cross-transmission of MRSA ST398 in the slaughterhouse.

We also observed that besides cross-transmission events occurring during the direct handling of animals, MRSA ST398 strains have also the ability to persist over time, since we could recover isolates that are probably related (72 SNPs) from the two different sampling periods.

### 3.6. Similarity between MRSA ST398 Isolates from the Slaughterhouse and Human Infection

A single ST398 MRSA isolate associated with human bloodstream infection was recovered from one Portuguese hospital in 2016 [68]. This strain was characterized by *spa* type t011, a *spa* type associated with ST398, and harbored SCC*mec* Vc (5C2 and 5). Its genome was aligned against the isolates from the slaughterhouse, using ISU926 ST398 strain as a reference. SNP analysis grouped the eight isolates from the slaughterhouse into two different clades, clade a including strains with *spa* type t108 and clade B containing isolates belonging to *spa* type t011. The nosocomial isolate grouped within clade B, and differed from slaughterhouse isolates within this cluster between 321 and 378 SNPs. The closest slaughterhouse isolates with nosocomial isolate were from animals and worker colonization, characterized by *spa* type t011 (with 321 to 340 SNPs differences), rather than the isolates from meat that had *spa* t108 and ranged from 368 to 378 SNPs (Figure 1).

Although MRSA ST398 from animals, slaughterhouse and hospital are not closely related, they shared a high number of antibiotic and virulence genes (Table S2). In particular, a total of 12 antimicrobial resistance genes and 48 virulence genes were common between the human clinical ST398 strain and the slaughterhouse strains.

Still, isolates from the two settings differed in genes conferring resistance to aminoglycosides (slaughterhouse: *aac(6′)-aph(2′′), aadD*, *str;* hospital: *ant(9)-Ia* gene) and each contained specific genes. While genes conferring resistance to erythromycin (*ermC*), streptogramin (*vga(A)LC*), chloramphenicol (f*exA*) were found only in slaughterhouse isolates from meat, animal, equipment, and operator; genes conferring resistance to pleuromutilin, lincosamide, and streptogramin A (*lsa(E)*) lincosamide (*lnu(B*) and trimethoprim (*dfrG*) were present only in the hospital isolate. Additionally, only strains from slaughterhouse carried the zinc metalloproteinase aureolysin (*aur*), the adhesin (*sdrE*) and the clumping factor (*clfA*, *clfB*) (Table S2).

## 4. Discussion

This study provided for the first time clear evidence for dissemination to humans of MRSA ST398 from the pig production chain in a Portuguese slaughterhouse. We showed that MRSA were disseminated not only in live pigs at the entrance of the slaughterhouse, but also in the entire environment including equipment, meat, and workers. The pig contamination with MRSA probably occurred in the farm setting, due to animal-to-animal transmission promoted by their usual contact and also with surrounding environment at production level and, afterwards, during transport, before entering the slaughterhouse. This is supported by the finding in our studies of very similar isolates in different animals both by PFGE and SNPs analysis and by a previous study wherein a massive occurrence of ST398-MRSA (99%) in two independent Portuguese swine farms was found [24]. Additionally, all the isolates lacked the IEC, a cluster related to the adaptation of the bacteria to non-human animal hosts [19], further confirming the pig origin of the ST398 isolates. Transmission was also observed between live pigs and workers, between equipment and meat and between meat and the workers as supported by the minor distance in the SNPs analysis performed and similar antimicrobial resistance and virulence profiles observed. The colonized workers and the pork samples, which are one of the final products that reach consumers, may then serve as a source for the transmission of the MRSA in the community. To confirm this hypothesis, sampling of the resultant meat products at the supermarket, and consumers manipulating these meat products should be performed in the future.

The selection of the best SNP difference cut-off between isolates to establish the occurrence of transmission events remains a major challenge for WGS-based surveillance due to different factors that can influence this parameter, namely the diversity, size and type of the bacterial population analyzed, the time period considered and strain mutation rate. The SNPs cut-off used in this study was previously validated for a situation of a MRSA nosocomial outbreak [65], different from that of dissemination in slaughterhouses. For that reason, some ambiguity can be associated with certain epidemiological links established.

MRSA ST398 strains with few SNPs difference (72 SNPs) were collected from two different sampling periods, suggesting persistence of MRSA strains within the slaughterhouse. Persistence could be related to the formation of biofilms or to resident microbiota of the slaughterhouse. However, to confirm this, further studies would have to be conducted to estimate a possible SNPs cut-off for persistence in the slaughterhouse environment. Our data also imply that disinfection procedures in place are probably ineffective given that both equipment and hands of workers were contaminated with MRSA before and after disinfection. A possible explanation is the presence of resistance genes for biocides found in a great proportion of the MRSA isolates analyzed in this study, which probably contributed to the MRSA persistence observed.

We found a high prevalence of ear and rectum MRSA carriage in live pigs (60%). The prevalence of MRSA detected in this study is much higher than the rates reported in other European countries namely in Italy (38%) [66], Denmark (13%) [69], Switzerland (1.3%) [70], and at the German–Dutch border (21%) [71]. Conversely, this prevalence is lower than the frequency reported in a recent study performed in healthy pigs in farms in Portugal (99%) [24]. The differences in prevalence probably are explained by the fact that pigs originated from different farms, with different sizes and procedures. On the other hand, the high prevalence of MRSA carriage in live pigs in Portugal might be either a result of beta-lactam usage pressure in animal production, due to the importation of pigs already colonized with MRSA [24] or to co-selection due to the heavy metal use as feed supplements [72]

Despite the fact that we clearly observed cross-transmission of isolates between the different players in the slaughterhouse, we could identify as many as 14 genetically related PFGE subtypes, revealing a relatively high heterogeneity of the bacterial collection (Figure S1), all belonging to ST398 and *spa* types t108, t011 and t1451, as previously described [73]. A high heterogeneity within German and Dutch ST398 isolates was reported before [74]. The origin of such genetic diversity is probably related to the ability of strains of this clonal type to exchange mobile genetic elements [75], which is shown in our study by the multiple antibiogram types and different virulence gene profiles found in the collection analyzed. However, to confirm this, deeper and more complete genomic analysis would have to be done.

All MRSA exhibited a multidrug resistance phenotype and the most predominant resistance profile found exclusively in live pigs at the slaughterhouse entrance included resistance to penicillin, oxacillin, cefoxitin, erythromycin, clindamycin, tetracycline, and chloramphenicol. It was reported that *S. aureus* multidrug resistant strains during slaughtering process have been increasing dramatically [76]. Most of these antibiotics (tetracycline, penicillin, erythromycin and clindamycin) are commonly used in veterinary medicine in Portugal [77] which could explain the high resistance levels to these drugs detected in MRSA isolated in this study. Furthermore, the prevalence of biocide resistance genes in the present MRSA ST398 collection was very high namely for *mepA*, *sepA*, *norA*, *lmrS,* and *qacAB*. Biocides, including QACs, are also extensively used in animal husbandry [78]. They are applied as antiseptics to treat minor skin animal injuries and as disinfectants of surfaces and worker’s hands, to maintain the required levels of hygiene at farms, slaughterhouse and food-processing premises [78]. The carriage of *qacC* could thus be an advantageous bacterial trait not only during colonization of animals but also during environmental and human colonization, and thereby contribute to the selection and spread of MRSA ST398. QACs have been found to collocate with antibiotic resistance genes on plasmids and chromosomal mobile genetic elements [79]. Thus, the widespread usage of QACs for disinfection in slaughterhouse and farm animal operations might contribute to the co-selection or co-retention of antibiotic resistance in slaughterhouse environment, which makes the eradication of MRSA ST398 more difficult. On the other hand, the presence of efflux pumps (*lmrs*, *sepA*, *mepA*, *norA* and *qac*) appeared to be associated with quinolone resistance in our study, a phenomena that can be explained by the fact that some efflux pumps for biocides can additionally transport quinolones [38]. The fact that there is a higher prevalence of quinolone resistance in meat, when compared to other sample types might be related to the fact that meat is rich in iron, a metal that was found to alter the expression of NorA in *S. aureus* [80].

Besides being multidrug resistant, ST398 MRSA isolates carried several virulence genes (*hlb*, *hlg*, *sel*, *sep,* and *sea*). The great majority of the ST398 isolates analyzed so far carry hemolysin-encoding genes [14] however, low levels of enterotoxin occurrence have been reported for MRSA ST398 isolates from Germany and France [14,81]. All the isolates lacked some clinically important *S. aureus*-associated virulence determinants, such as leukotoxins, toxic shock syndrome toxin, ACME and PVL, which is in line with the results of other studies focused on pig population [19] where PVL have been detected at a low frequency in pig isolates [1].

The first invasive MRSA ST398 described among Portuguese hospitals [82], included in this study, shares characteristics with the slaughterhouse isolates and with isolates collected in hospital settings across Europe [83,84], namely *spa* type t011 and SCC*mec* V, IEC negative, suggesting an animal origin of this isolate. Although this isolate was collected in the same region and time period as the slaughterhouse isolates, comparison of their genomes by SNPs analysis showed that they are probably not closely related (321–378 SNPs), with also different antibiotic resistance and virulence profiles. Given the high rates of MRSA ST398 in pigs in Portugal, surveillance should be reinforced in order to monitor the eventual spread of MRSA ST398 in the Portuguese hospitals, where EMRSA-15 has massively dominated since the beginning of 2000 [85].

## 5. Conclusions

These results suggest that pig colonization at slaughterhouse entrance, the development of resistance to biocides in use on the hygienic environmental programs are potential risk factors for meat contamination and dissemination of MRSA ST398 to humans in Portugal. To better understand if MRSA ST398 are emerging as pathogens in Portugal, it will be fundamental to increase MRSA surveillance in hospitals and community.

## Supplementary Materials

The following are available online at https://www.mdpi.com/2076-2607/8/12/1892/s1, Table S1: Phenotypic and genotypic characterization of 32 MRSA ST398 isolated from slaughterhouse and hospital based on PCR amplification, Table S2: Virulence and antimicrobial resistance determinants of 9 MRSA ST398 isolated from slaughterhouse and hospital based on WGS, Figure S1: Pulsed-field gel electrophoresis (PFGE) typing of MRSA ST398 isolates. The dendrogram showing the clustering of strains was performed by the analysis of PFGE profiles with the BioNumerics software, with 1.25% tolerance and 0.5% optimization. PFGE types and subtypes defined by groups formed at 80% and 95% Dice similarity cutoffs, respectively.
