# Supplementary figures and images for: Evidence for the Dissemination to Humans of Methicillin-Resistant Staphylococcus aureus ST398 through the Pork Production Chain: A Study in a Portuguese Slaughterhouse
Source: Microorganisms. 2020 Nov 29;8(12):1892. doi: 10.3390/microorganisms8121892 (PMC7759831; doi:10.3390/microorganisms8121892)

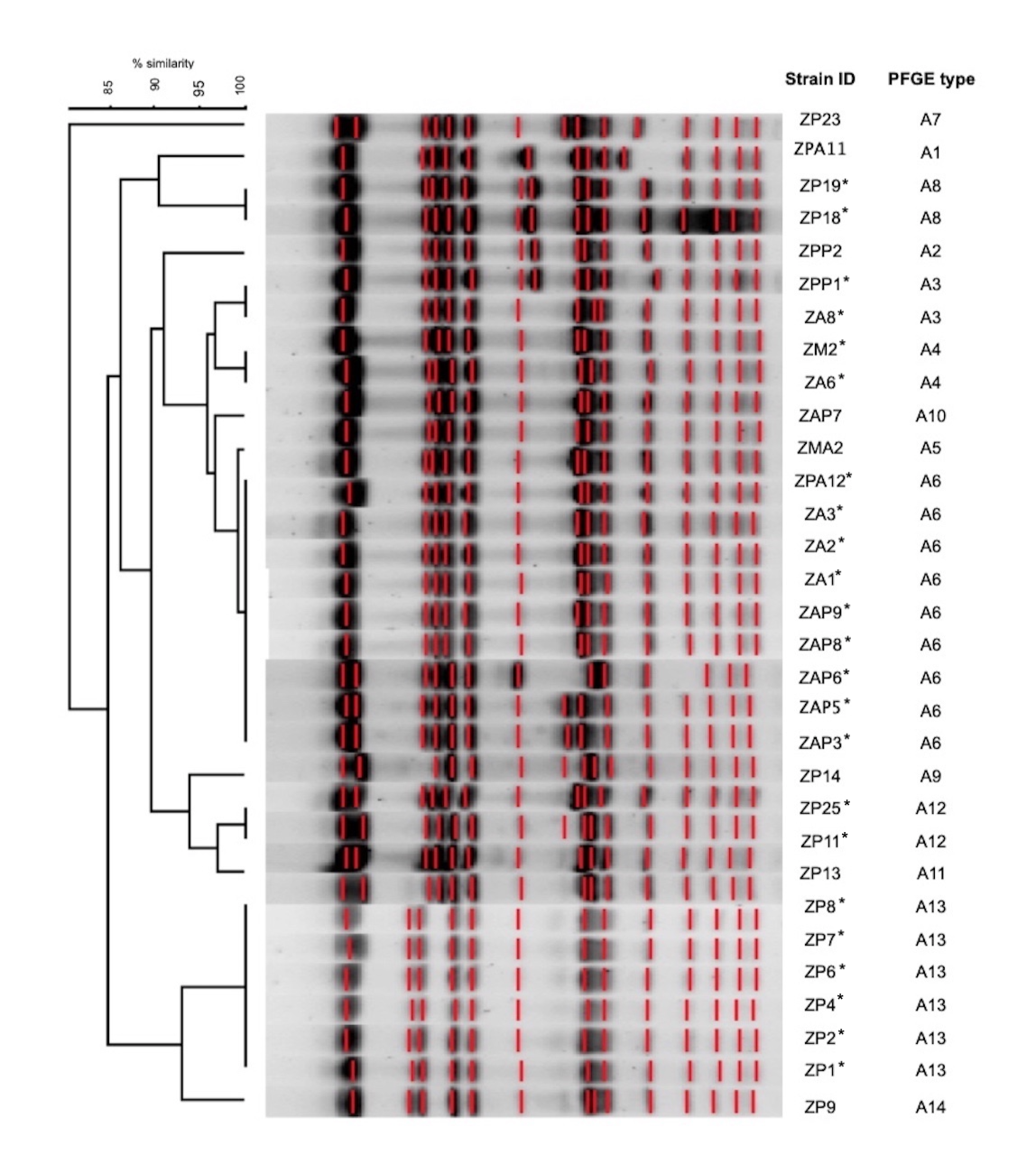

Supplement: Supplementary file 1 [file microorganisms-08-01892-s001.zip › Supplementary_Materials_27Nov2020/OBouchami_MRSA_Figure S1_27Nov2020.tiff]
